# Supplementary material for: Effect of 5-Azacitidine Treatment on Redox Status and Inflammatory Condition in MDS Patients
Source: Antioxidants (Basel). 2022 Jan 9;11(1):139. doi: 10.3390/antiox11010139 (PMC8773071; doi:10.3390/antiox11010139)
Supplement: Supplementary file 1 [file antioxidants-11-00139-s001.zip › antioxidants-1520387-supplementary.pdf]

Supplementary Table S1. Clinical features of the patients enrolled in the study.

PATIENTS	% BLASTS in BM	TIME FROM DIAGNOSIS TO ANALYSIS	WHO-2016	IPSS-R	IPSS-R RISK	CYTOGENETIC RISK	MUTATIONAL PROFILE	SCORE MUTATIONAL	MOLECULAR RISK	TREATMENT
**1**	3	1 month	MDS-SLD (Early Stage)	BR	Low Risk	Good (Favorable)	***ASXL1*** (37%), *U2AF1* (41.8%), *SETBP1* (2.9%)	3	High Risk	Azacitidine
**2**	2	4 month	MDS-SLD (Early Stage)	VL	Low Risk	Good (Favorable)	***ASXL1*** (44,2%), *U2AF1* (40,85%)	2	High Risk	NA
**3**	2	3 month	MDS-RS-SLD (Early Stage)	BR	Low Risk	Good (Favorable)	*SF3B1* (43,2%), *TET2* (47,2%)	2	Low Risk	NA
**4**	2	1 year	MDS-MLD (Early Stage)	INT	High Risk	Good (Favorable)	***TP 53*** (28,6%), ***ASXL1*** (25,4%), *SRSF2* (28,6%), *U2AF1* (30,6%)	4	High Risk	Azacitidine
**5**	0	5 month	MDS-MLD (Early Stage)	VL	Low Risk	Good (Favorable)	***ASXL1*** (18,3%), ***EZH2*** (37,3%), ***RUNX1*** (9,9%), *TET2* (28%)	4	High Risk	Azacitidine
**6**	1	<1 month	MDS-MLD (Early Stage)	INT	High Risk	Poor (Unfavorable)	***TP53*** (76,4%)	1	High Risk	NA
**7**	3	1 year	MDS-MLD (Early Stage)	VL	Low Risk	Good (Favorable)	*IDH2* (46,6%), *DNMT3A* (45%)	2	Low Risk	NA
	1	1.5 years					*IDH2* (46.7%), *DNMT3A* (46.5%)	2	Low Risk	Azacitidine
**8**	2,5	1 year	MDS-MLD (Early Stage)	VL	Low Risk	Very Good (Favorable)	ND	ND	Low Risk	Supportive care
**9**	3	2 years	MDS-MLD (Early Stage)	VL	Low Risk	Good (Favorable)	Not detected	0	Low Risk	NA
**10**	2	5 month	MDS-MLD (Early Stage)	BR	Low Risk	Good (Favorable)	***ASXL1*** (12,5%)	1	High Risk	Supportive care
**11**	3	2 year	MDS-RS-MLD (Early Stage)	BR	Low Risk	Good (Favorable)	ND	ND	Low Risk	Supportive care
**12**	4	2 years	MDS-RS-MLD (Early Stage)	VL	Low Risk	Good (Favorable)	***RUNX1*** (49.3%), *SF3B1* (28%)	2	High Risk	NA

**13**	1	8 month	MDS *del*(5q) (Early Stage)	INT	High Risk	Good (Favorable)	Not detected	0	Low Risk	Supportive care
**14**	3	<1 month	MDS *del*(5q) (Early Stage)	BR	Low Risk	Good (Favorable)	**ASXL1** (47.7%), TET2 (47.3%)	2	High Risk	NA
**15**	2	1.5 month	MDS SA (Early Stage)	BR	Low Risk	Good (Favorable)	***RUNX1*** (18.8%), *SF3B1* (42%), *JAK2* (5.8%), *TET2* (40.9%)	4	High Risk	Supportive care
**16**	6	1.5 month	MDS EB-1 (Advanced Stage)	INT	High Risk	Poor (Unfavorable)	*SF3B1* (40,3%)	1	Low Risk	NA
**17**	6	1 year	MDS EB-1 (Advanced Stage)	AR	High Risk	Intermediate (Unfavorable)	***ASXL1*** (32.5%), *ZRSR2* (67.4%)	2	High Risk	Supportive care
**18**	9	<1 month	MDS EB-1 (Advanced Stage)	INT	High Risk	Good (Favorable)	***ASXL1*** (11,6%)	1	High Risk	Azacitidine
**19**	6	<1 month	MDS EB-1 (Advanced Stage)	INT	High Risk	Good (Favorable)	*WT1* (11.8%)	1	Low Risk	NA
	1	4 month					*WT1* (17.4%)	1	Low Risk	Azacitidine
**20**	5,5	<1 month	MDS EB-1 (Advanced Stage)	MAR	High Risk	Very Poor (Unfavorable)	***TP53*** (2.9%), ***RUNX1*** (1.9%)	2	High Risk	NA
**21**	18	<1 month	MDS EB-2 (Advanced Stage)	INT	High Risk	Good (Favorable)	***ASXL1*** (31.1%), *FLT3* (9.3%), *SRSF2* (32.8%), *TET2* (33.3%), *NRAS c.35G>A* (11.8%), *NRAS c34G>A* (5.9%), *ETV6* (2.5%)	7	High Risk	NA
	0	3 month					***ASXL1*** (18%), *FLT3* (1%), *SRSF2* (21.1%), *TET2* (42.7%), *NRAS c.35G>A* (3.9%), *NRAS c34G>A* (1.1%), *ETV6* (3.3%), *CEBPA* (2.7%), *JAK2* (16.4%), *CBL* (10%)	10	High Risk	Chemotherapy
**22**	12	2 years	MDS EB-2 (Advanced Stage)	MAR	High Risk	Intermediate (Unfavorable)	***ASXL1*** (45,9%), ***RUNX1*** (48%), ***EZH2*** (94,7%), *NRAS* (11,5%), *FLT3* (2%), *CEBPA* (1,7%)	6	High Risk	Azacitidine
**23**	18	<1 month	MDS EB-2 (Advanced Stage)	MAR	High Risk	Very Poor (Unfavorable)	***TP53*** (70.5%)	1	High Risk	NA

**24**	15	<1 month	MDS EB-2 (Advanced Stage)	AR	High Risk	Good (Favorable)	***ASXL1*** (41.6%), ***RUNX1*** (47.3%), ***EZH2*** (93.3%), *CSF3R* (45.1%), *CSF3R* (5.9%)	5	High Risk	NA

Note: BM: Bone Marrow; Myelysplastic Syndrome (MDS); MDS with single lineage dysplasia: MDS-SLD; MDS with multilineage dysplasia: MDS-MLD; MDS with ring sideroblasts: MDS-RS; MDS with SLD and ring sideroblasts (MDS-RS-SLD); MDS with MLD and ring sideroblasts (MDS-RS-MLD); MDS with isolated del(5q): MDS (*del*5q); MDS EB-1, -2: MDS with Excess of Blasts-1, -2.
IPSS-R: Revised International Prognostic Scoring System. **Low Risk** (Very Low Risk (VLR)/Low Risk (LR); **High Risk** (Intermediate Risk (INT)/High Risk (HR)/Very High Risk (VHR).
Cytogenetic risk: Favorable [Very good (-Y, del(11q), Good (normal, del(20q), del(5q) alone or with 1 other anomaly and del(12p)]; Poor [Poor (complex with 3 abnormalities, der(3q) or chromosome 7 abnormalities), Very poor (complex with ≥3 abnormalities)]; Intermediate (all other single or double abnormalities not listed). **Favorable** (Good karyotypes) vs. **Unfavorable** (Intermediate, Poor and Very Poor karyotypes).
Mutational Score: number of total somatic mutations detected in each patient. In Mutational Profile, the Variant Allelic Frequencies (VAFs) are shown in parentheses and genes indicated in bold are High Molecular Risk (HMR) genes [5].
Molecular risk: High Risk (presence of at least one mutation in any of High Molecular Risk (HMR) genes) vs. **Low Risk** (absence of mutations in HMR genes).
NA: Not Applicable; ND: No Data.
